# Shopping for truth pluralism

**DOI:** 10.1007/s11229-020-02792-z

**Published:** 2020-07-22

**Authors:** Will Gamester

**Affiliations:** School of Philosophy, Religion and History of Science, University of Leeds, Leeds, West Yorkshire UK

**Keywords:** Truth pluralism, Ontological pluralism, Mind-dependence, Douglas Edwards, Michael Lynch

## Abstract

Truth pluralists say that the nature of truth varies between domains of discourse: while ordinary descriptive claims or those of the hard sciences might be true in virtue of corresponding to reality, those concerning ethics, mathematics, institutions (or modality, aesthetics, comedy…) might be true in some non-representational or “anti-realist” sense. Despite pluralism attracting increasing amounts of attention, the motivations for the view remain underdeveloped. This paper investigates whether pluralism is well-motivated on ontological grounds: that is, on the basis that different discourses are concerned with different kinds of entities. Arguments that draw on six different ontological contrasts are examined: (i) concrete versus abstract entities; (ii) mind-independent versus mind-dependent entities; (iii) sparse versus merely abundant properties; (iv) objective versus projected entities; (v) natural versus non-natural entities; and (vi) ontological pluralism (entities that literally exist in different ways). I argue that the additional premises needed to move from such contrasts to truth pluralism are either implausible or unmotivated, often doing little more than to bifurcate the nature of truth when a more theoretically conservative option is available. If there is a compelling motivation for pluralism, I suggest, it’s likely to lie elsewhere.

## Introduction

Some facts are more suspicious than others. Most of us are happy to concede that there is a world out there, existing independently of what we happen to think about it, and that when we’re talking about tables, chairs, cats, mountains, and perhaps electrons, quarks, and so forth, what we’re trying to do is *describe* that world. But it’s hard to feel this way about everything we say. For one reason or another, it can be hard to see how the ethical, the mathematical, or the institutional, as well as the modal, the psychological, the aesthetic, the comedic, and so on, is a part of that world. And yet it’s often equally hard to believe that all such talk involves misapprehension—that it is an attempt to describe some aspect of the world that simply isn’t there.

One way to try and split the difference is to appeal to a non-representational theory of truth in the problematic domains. Suppose it is a mind-independently given fact that Felix, the cat, is furry and that Lexy, the electron, is negatively charged. If so, then (1) and (2) might be true in virtue of accurately representing, or “corresponding to”, those facts:

1. Felix is furry.
2. Lexy is negatively charged.
3. 7 is prime.
4. Kicking puppies is wrong.
5. Prince William is married.

But instead of appealing to a mathematical, ethical, or institutional fact to explain why (3), (4), or (5) is true, we might endorse some coherentist, pragmatist, or epistemic conception of truth. Perhaps (3), (4), and (5) are true in virtue of *cohering* with the relevant mathematical axioms, stable moral theory, or body of law. Most prominent in this context is Crispin Wright’s (1992: p. 48) generalisation of the notion of a mathematical proof: superassertibility. Roughly speaking, (3), (4), or (5) is assertible iff there is some state of information that warrants believing or asserting it; it is superassertible iff this warrant would survive no matter how much that state of information was improved.^1^ The result is a kind of *truth pluralism*: what it is for something to be true varies between domains.^2^

Many, including me, have found this picture intuitive enough to spend considerable time and energy trying to figure out how the theory is best formulated and how to respond to objections. Comparatively little, however, has been done to investigate the motivations for truth pluralism—to see if the foundations are secure, before trying to build elaborate structures and fend off attacks.^3^

There is good reason to think that we need more than the intuitive rationale just given. When it comes to vindicating our competing “realist” and “anti-realist” intuitions in different domains, there is no shortage of other contenders. Moreover, if one’s motivation is merely suspicion about the relevant entities and facts, then it’s not obvious that truth pluralism alone gets you what you want. If (3) is true *at all*, then by any plausible logic it follows that there is something that is prime (namely, the number 7). To that extent, at least, there is a mathematical object. Similarly, it follows that there is something that 7 is (namely, prime)—so to that extent there is a mathematical property. And if there is a mathematical object that exemplifies a mathematical property then there is, in that sense, a mathematical fact. So, while we may not have appealed to mathematical entities to explain why (3) is true, it’s not obvious that we’re off the hook just for that.^4^

The most important demand for more motivation, however, comes from the fact that the pluralist’s place in the explanatory story makes for an uncomfortable stopping point. That is, even if we *grant* that the nature of truth varies between domains, the obvious question is: Why? Why is it that truth is a matter of corresponding to the facts in some domains, but is a matter of coherence or superassertibility in others? What is it about mathematics, ethics, or the institutional, in contrast to, say, the hard sciences, that makes discourse concerning the former apt for one truth property, while the latter is apt for another? Are we to take the difference as brute? Explanation must stop somewhere, but stopping here would surely be unsatisfying.

Two broad strategies present themselves. The first is metaphysical or ontological. It appeals to differences in the nature of the entities—objects, properties, or facts, say—that different discourses are concerned with; contrasting, say, the medium-sized dry goods of everyday life with abstract objects like numbers, prescriptive properties like wrongness, or mind-dependent properties like being married. The second is functional or teleological. It appeals to differences in what the relevant thought and talk is *for*; in particular, that while some discourses serve a representational function—meaning we use the very referring terms of the discourse to explain its existence—others serve a non-representational function.^5^

The strategies are by no means exclusive.^6^ Misgivings about the entities of some domain might motivate a non-representational explanation of the purpose of the discourse; a non-representational explanation of the discourse might motivate a different conception of the relevant entities. And different strategies might be better suited to different domains: the metaphysical approach to mathematics, and the teleological to ethics, say.

Establishing that there are substantive metaphysical or teleological differences between domains—perhaps with accompanying epistemological or normative^7^ differences—is, I’ll assume, sufficient to render truth pluralism a salient theoretical option, a live possibility worth taking seriously. But presumably the pluralist thinks it does more than *that*. The pluralist’s view is that such differences give us some reason to *prefer* truth pluralism to its theoretical competitors, and in particular to global versions of the theories the pluralist endorses only locally.

This paper sets out to see whether this is so, focusing on the metaphysical strategy in particular. This narrowing of focus has tactical motivation. It is partly pragmatics: we can only cover so much in one paper, and this approach provides plenty to engage with. But it is also rhetorical. The metaphysical strategy has been more prominent in the literature,^8^ but I strongly suspect the pluralist is better served by the teleological strategy. By detailing the challenges facing the former, I hope to refocus pluralists’ efforts.

Any metaphysical motivation for truth pluralism will face a flat-footed challenge. Granted, different discourses might be concerned with radically different kinds of entities. But if we’re happy with the idea that truth at least sometimes consists in correspondence with the facts, then why not think that it always does so, and that what varies is simply the kinds of facts that different discourses correspond to? If we can have variation in the entities or facts—in the “worldly” relatum of the correspondence relation—then why *also* think that we need variation in the nature of truth? This kind of argument has sometimes been pressed as the “double-counting” or “Quine-Sainsbury” objection to pluralism.^9^

I think the best way to view the metaphysical strategy is an attempt to meet this challenge by arguing that ontological variation of one kind or another *motivates* truth pluralism. The arguments for pluralism we’ll consider below thus exhibit a common structure, with two core claims. *First*, that there is an important kind of ontological variation between the entities of different discourses. As we’ll see, these range from the familiar (some entities are mind-independent, others mind-dependent; some are concrete, others abstract) to the highly contentious (the nature of existence varies between different entities). *Second*, that—given certain auxiliary premises—truth pluralism follows: in particular, that truth should be understood representationally in discourses concerning entities of one kind, and non-representationally in discourses concerning entities of another. While the plausibility of the first step is, of course, crucial to the overall pluralist programme, in the spirit of open-minded generosity we’ll mostly grant to the pluralist that there are substantive ontological differences, of one kind or another, between domains. Our primary focus will thus be the second step: whether such differences give us any reason to prefer pluralism to its theoretical competitors.

Objects and properties vary along countless dimensions, which means the scope for finding an ontologically-driven pluralism of this form is vast. To focus things, we’ll draw on the pioneering work of Douglas Edwards (Sect. 2) and Michael Lynch (Sect. 3).^10^ As motivation for pluralism, we’ll consider that certain entities: (i) literally exist in a different way; (ii) are projected; (iii) are merely abundant; (iv) are mind-dependent; (v) are non-natural; and (vi) are abstract. (i)–(iii) are the focus of Sect. 2. We consider them as grounds for Edwards’s claim that what is so is sometimes *grounded in* what is true, and thus that truth is sometimes non-representational. (iii)–(vi) are discussed in Sect. 3. There, we consider Lynch’s argument that (a) representational or correspondence theories of truth should be cashed out in causal-representational terms, (b) causal representationalism does not apply in certain discourses, and therefore (c) that truth should be understood non-representationally in such discourses. We consider (iii)–(vi) as grounds for (b).

We hereby survey a wide variety of ontologically-driven arguments for pluralism. None, I’ll argue, is compelling. Even when the ontological variation in question is highly contentious, the auxiliary premises needed to move from such a claim to truth pluralism are either implausible or unattractive—sometimes only serving to bifurcate the nature of truth when there is, by the pluralist’s own lights, a more theoretically conservative option available. We thereby vindicate the “double-counting” worry that the metaphysical strategy renders truth pluralism something of a theoretical spinning-wheel (while leaving open that the theory may find more compelling motivation elsewhere).

## Being grounded in truth?

Consider the T-schema:

(T): ‘*p*’ is true iff *p*

One way of trying to argue for truth pluralism on ontological grounds is to argue that, due to the nature of the relevant entities, while certain instances of (T) need to be read with a left-to-right order of explanatory dependence (e.g. ‘iron is magnetic’ is true *because* iron is magnetic), other instances need to read with a right-to-left order of explanatory dependence (e.g. motorbikes are cool *because* ‘motorbikes are cool’ is true). In the former cases, what is true depends on what is so, and thus—the reasoning goes—we ought to endorse a representational conception of truth. However, in the latter cases, what is so depends on what is true; so our account of what it is for such sentences to be true cannot be representational, on pain of circularity. If we’re to give a substantive account of what it is for such sentences to be true, then, truth must be understood non-representationally.

This is the strategy that has been developed, in a couple of different ways, by Douglas Edwards. As we’ll see, the crucial element of this argumentative structure is in arguing for the right-to-left explanatory reading of certain instances of (T). Edwards is hereby taking up an anti-realist tradition, which maintains that, in certain domains, what is so depends in an important sense on what we say or think is so (or on our social practices more broadly); that the distinctions we’re drawing are not an antecedently given part of the mind-independent world, but instead created by us.

Nonetheless, this strategy runs into a recurrent difficulty. The problem is that the peculiarly *alethic* part of this explanatory story—which says that what is so depends on the *truth* of certain sentences (propositions, beliefs)—looks redundant. It is quite possible to think that the coolness of motorbikes (say) depends on us and our social practices without thinking that it depends specifically on the truth of the sentence ‘motorbikes are cool’. Indeed, I’ll argue that any such explanatory story that incorporates an alethic element has a more economical counterpart that does not. But without the alethic element of the story, the argument for truth pluralism cannot get up and running: we can endorse the left-to-right explanatory reading of the T-schema across the board. Truth pluralism thus becomes a theoretical spinning-wheel.

That’s the general dialectic that we’ll see play out below. Now for details.

### On ontological pluralism

Let’s start with an argument developed jointly by Edwards and Aaron Cotnoir (Cotnoir and Edwards 2015). The ontological variation Cotnoir and Edwards (2015: pp. 119–120) discuss is the most radical variety going: a kind of ontological pluralism which says that the very nature of existence varies between different entities. In particular, for concrete entities like tennis balls or the Eiffel Tower, it is captured by what they call “Alexander’s Dictum” (AD), while for abstract entities like numbers, it is captured by the “Neo-Fregean Principle” (NFP):

(AD): To exist is to have causal powers
(NFP): To exist is to be the referent of a singular term that appears in a true sentence

Granting ontological pluralism, one can readily get a sense of how the argument for truth pluralism is going to go. The basic idea is that what is true is sometimes grounded in what exists—i.e., when the relevant entities exist in the sense of (AD)—but what exists is sometimes grounded in what is true—i.e., when the relevant entities exist in the sense of (NFP). Truth is thus sometimes representational (in the former cases), and sometimes non-representational (in the latter cases). I’ll run through this in more detail in a moment.

However, Cotnoir and Edwards in fact formulate their argument in a curious way. Calling the kind of existence captured by (AD), BEING_1_, and the kind of existence captured by (NFP), BEING_2_, their argument for truth pluralism (2015: p. 128, lightly edited) relies on the idea that these two kinds of existence are *equi*-*fundamental*:

**Table:** 

(I)	BEING_1_ and BEING_2_ are equi-fundamental	Premise
(II)	BEING_1_ grounds TRUTH_*i*_	Premise
(III)	TRUTH_*j*_ grounds BEING_2_	Premise
(IV)	TRUTH_*i*_= TRUTH_*j*_	Assumption for reductio
(V)	BEING_1_ grounds BEING_2_	From (II), (III), (IV), transitivity of grounding
(VI)	If *x* grounds *y*, then *x* is more fundamental than *y*	Definition of ‘grounding’
(VII)	BEING_1_ is more fundamental than BEING_2_	From (V), (VI)
(VIII)	*Contradiction*	From (I), (VII)
(IX)	TRUTH_*i*_ ≠ TRUTH_*j*_	Reductio from (I)–(VIII)

The rationale for premise (III) is just (NFP): what exists is sometimes grounded in what is true. Assumption (IV) is truth monism: the view that the nature of truth is uniform. The rationale for premise (II) takes some reconstruction. Recall (1):

(1): Felix is furry

If things like cats exist in the sense of (AD), then Felix’s existence presumably does not depend on the truth of (1). It’s thus natural to think that (1) is true at least in part *because* Felix exists—truth comes after existence in the explanatory order of things. If that’s right, then the truth of (1) is grounded in BEING_1_, as premise (II) states. Granting that, and given the equi-fundamentality premise (I), it’s easy to see how the truth monist is led into contradiction.

The argument is neat, but unfortunately including premise (I) renders the overall picture here unstable. We’re left with a straightforward dilemma. (NFP) accounts for the existence of certain entities in terms of certain true *sentences*. If these sentences are to be true, then *they* must exist. But what kind of existence do they have? If BEING_2_, then we’re off on a vicious regress. If, however, the regress stops because some relevant sentence has BEING_1_, then BEING_2_ is grounded in BEING_1_ after all, violating (I).^11^

Fortunately, it seems that the equi-fundamentality premise is not only problematic but unnecessary, as far as the argument for truth pluralism is concerned. Suppose that the above reasoning for premise (II) is correct, so the truth of (1) is grounded in the existence of Felix, as per (let’s grant) a representational conception of truth. Now recall (3):

(3): 7 is prime

If things like numbers exist in the sense of (NFP), then 7’s existence is grounded in its being the referent of a singular term in a true sentence. The theory does not tell us *which* true sentence(s), but suppose for the sake of argument that it’s (3) (or that (3) is among them).^12^ We thus cannot explain the truth of (3) by appealing to the number 7 and what it is like, on pain of circularity. Therefore, in this instance truth arguably cannot consist in accurate representation of an antecedently given reality. So—the pluralist reasons—it must consist in something else, something non-representational. It is consistent with this argument that (3) itself has BEING_1_, and thus that all BEING_2_ is ultimately grounded in BEING_1_. (Or so it seems, and I’m willing to grant for the sake of argument.)

There is, however, a deeper worry than any concerning the details of the argument itself. Recall that, for the ontological pluralist, (NFP) only tells us what existence consists in for certain entities—just to have a name, call them *the constructed entities*. On pain of circularity, it follows that truth cannot be representational for those sentences whose truth grounds the existence of a constructed entity. (That’s the argument for truth pluralism.) So, for such sentences, let’s suppose that truth consists in some non-representational property, like superassertibility. It must follow that, for any constructed entity *x*, *x* exists iff *x* is the referent of a singular term that appears in a sentence *that is superassertible*. But now we face a challenge. For given that we are committed to the truth of this biconditional, why not go in for (NFP*) instead of (NFP) as our account of what it is for a constructed entity to exist?:

(NFP*): To exist is to be the referent of a singular term that appears in a superassertible sentence

If we endorse (NFP*) instead of (NFP), then while the existence of the number 7 still depends on (3) being superassertible, the *truth* of (3) can in turn depend on the existence of the number 7. Thus, even while the nature of existence varies, what is true can be uniformly grounded in what exists. Truth can thus be representational across the board.

The question, then, is what could motivate (NFP) over its rival. The underlying ontological pluralism remains just as radical. The principal difference is just that (NFP) engenders a bifurcation in the nature of truth where (NFP*) does not. Theoretical economy demands the simpler option.

One possible objection to (NFP*) is that other sentences, like those concerning the furriness of cats, can be superassertible (or whatever) without the relevant entities existing. But *both* sides agree that superassertibility is sometimes sufficient for existence (numbers), but sometimes not (cats). That, we’re supposing, is a consequence of the ontological pluralism. The difference is just that the advocate of (NFP) maintains that it is only when truth reduces to superassertibility that the latter is sufficient for existence, while the advocate of (NFP*) maintains that this gets the order of explanation the wrong way around: it is because superassertibility is only sufficient for existence in some cases that in *those* cases the sentences wind up being true. Again, the upshot of doing things this way is that truth remains uniform, to (NFP*)’s credit. Nor will it help to say that the truth of (3) *entails* 7’s existence, whereas its superassertibility does not. The game is metaphysics, and as a matter of metaphysical necessity, both sides think (3) being superassertible is sufficient for 7’s existence. As a matter of conceptual necessity (or similar), the observation isn’t pertinent.

So, even granting that the patched version of Cotnoir and Edwards’s argument works, truth pluralism comes across as a spinning wheel in the ontological pluralist’s theoretical machinery, as predicted. Now, perhaps we could go back-and-forth on (NFP) and (NFP*) some more (though I’ve been unable to conjure up anything convincing on (NFP)’s behalf myself). At this point, however, I think we’re better served by looking for less controversial starting points.

### Edwards’s “strong” argument

#### On objective and projected properties

To provide an ontological foundation for truth pluralism, one need not go so far as to say that the very nature of existence varies. Less controversial is the idea that entities, which may all exist in the same way, come in importantly different varieties: while some are concrete, others are abstract; while some are mind-independent, others are mind-dependent. It is to such premises we now turn.

In his recent book, Edwards (2018) provides a “strong” argument for truth pluralism on the grounds that there are importantly different kinds of properties.^13^ Focusing on (utterances of) sentences of the form ‘*a* is *F*’, and assuming that predicates like ‘is *F*’ refer to properties like *F*ness, Edwards (67) says that we can distinguish three options vis-à-vis the property referred to by any particular predicate: (a) it is *objective*; (b) it is *projected*; or (c) there isn’t one. (c) is an error-theoretical option that we’ll set aside.

What is it for a property to be objective or projected? Edwards (68) explicates the difference via the biconditional (P):

(P): The object referred to by ‘*a*’ falls under the predicate ‘*F*’ iff the object referred to by ‘*a*’ has the property referred to by ‘*F*’

A property, *F*ness, is objective iff there is a left-to-right order of explanatory dependence on (P): if *a* falls under the predicate ‘is *F*’, then it does so *because* it has the property of *F*ness. *F*ness is projected iff there is the opposite direction of explanatory dependence: *a* has the property of *F*ness, if it does, *because* it falls under the predicate ‘*F*’. So the property of *being magnetic* is objective iff things fall under the predicate ‘is magnetic’ because they are magnetic; it is projected iff things are magnetic because they fall under the predicate ‘is magnetic’.

By this criterion, magnetism is objective: something’s being magnetic does not depend on us or which predicates we have. A property’s being objective is hereby supposed to capture a kind of realism. Edwards’s conception of projected properties, by contrast, taps into the anti-realist tradition mentioned above: that, at least sometimes, when we carve up the world, the categories we use do not latch onto distinctions that are already out there—limning nature at its joints—but instead *create* those distinctions.

Edwards goes on to argue that truth is representational when the sentence in question concerns an objective property; non-representational when it concerns a projected property. We’ll consider the argument below (Sect. 2.2.3). First, however, it’s important to consider the ontological distinction that constitutes the basis of the argument (Sects. 2.2.1, 2.2.2).

It is, as noted, highly plausible that there are objective properties. The reason that iron falls under the predicate ‘is magnetic’ is because iron is magnetic. Of course, this isn’t a *complete* explanation: we also need a metasemantic explanation of why ‘is magnetic’ refers to that property. After all, if we had used language differently, ‘is magnetic’ might have referred to the property of being invisible, and then iron would not fall under ‘is magnetic’, though it would still be magnetic. However, I’ve found it much more difficult to find a plausible example of a projected property in Edwards’s sense. This matters, since in Edwards’s own taxonomy any property that is not projected is objective; and so, by his own argument, truth for the relevant sentences is representational. Unless we have reason to think that some property is projected, Edwards’s argument for truth pluralism cannot get going.

A property, *F*ness, is projected iff any object *a* that is *F* is *F* because it falls under the predicate ‘is *F*’. The most plausible contender, I think, is the “self-referential” property: *falling under this predicate*. If any object falls under this predicate, then trivially it does so because it falls under this predicate; i.e., because it falls under ‘falls under this predicate’. But I doubt that there are any such objects, and thus no true instance of ‘*x* falls under this predicate’.

But what other property might be like this? Edwards’s (68) example is *being cool*:

One example here is the property of being cool: motorbikes have the property of being cool because motorbikes fall under the predicate ‘is cool’; rather than vice versa.

But this, I submit, is simply false. If motorbikes are cool, then what makes them cool is presumably the way they look, the sound they make, the freedom they give you, the associated clothing, the kind of person who rides them, that sort of thing. While I’m hardly an expert on the matter, I’m reasonably certain that falling within the extension of a predicate—even the predicate ‘is cool’—is not the kind of thing that makes something cool.^14^ Similar objections apply to Edwards’s other purportedly projected properties, which are primarily properties that are plausibly socially constructed; e.g. being the Governor of New York or being a woman (72–73).^15^

Now, one might concede the general point while still suggesting that falling under ‘is cool’ is *part* of what makes motorbikes cool. But why think this? For one thing, that there are other things that make motorbikes cool is necessary (motorbikes could hardly be cool *simply* in virtue of falling under the predicate); and once they are in place the other factors are sufficient (if they weren’t, then falling under the predicate could hardly tip the balance). There’s no explanatory work for falling under the predicate to do here. Alternatively, one might argue that the kinds of thing that explain why motorbikes are cool do so *by* explaining why they fall under the predicate ‘is cool’ (perhaps because they explain why ‘motorbikes are cool’ is true), which *in turn* explains why they are cool. This looks coherent, but it’s difficult to see what independent motivation it has. The additional cogs in the theoretical machinery are redundant.

It’s worth stressing that what I am querying here is Edwards’s *particular* conception of projection—the one on which his argument for truth pluralism rests—which explains property exemplification in terms of predicate satisfaction. Nonetheless, an anonymous referee worries that I am begging the question against ‘broadly response-dependent (or other forms of non-realist metaphysics)’ of properties like coolness, according to which what is cool depends, in some sense, on what we judge or say is cool, or on our social practices more broadly. This is a concern worth taking seriously. One may suspect that there could be some anti-realist metaphysics of properties like coolness that entails that they are projected in Edwards’s sense; and thus, even if we’re not convinced by Edwards’s example, projection may stand on firmer ground than it appears here. On the contrary, however, it is quite possible to subscribe to an anti-realist metaphysics of *F*ness without thinking that *F*ness is projected; indeed, there is good reason to think that any anti-realist metaphysics of *F*ness that does entail that *F*ness is projected ought to be replaced by another, closely-related, metaphysics that does not. Let me explain.

Suppose that *F*ness is projected, so any object that is *F* is so because it falls under ‘*F*’. Now, let ‘φ’ abbreviate whatever conditions need to be satisfied to fully explain why some object *a* falls under ‘*F*’. Since *F*ness is *ex hypothesi* projected, we know that φ does not mention that *a* is *F*. We also know that it does not mention that *a* falls under ‘*F*’, on pain of circularity. But beyond that, φ can build in pretty much whatever conditions you like. That is, it may well be that what falls under ‘*F*’ depends, in some sense, on what we judge or say is *F*, or on our social practices more broadly. It can, for instance, include that certain subjects would have certain responses to *a* in certain circumstances. To make the point explicit: we can build into φ whatever anti-realist story about properties like coolness one prefers.

Now, if *a* falls under ‘*F*’, then (by disquotation) *a* is *F*. And if φ holds then *a* falls under ‘*F*’. Therefore, if φ holds, then *a* is *F*; that is, φ is sufficient for *a*’s being *F*. However, since φ does not mention that *a* falls under ‘*F*’, and *F*ness is projected, it follows that φ is not *explanatorily* sufficient for *a*’s being *F*. We’re thus led to the view that, despite φ’s being sufficient for *a*’s being *F*, we have to add the fact that *a* falls under ‘*F*’ to φ—call this, φ+—before we can fully explain why *a* is *F*.

But now, I hope it is clear, there is a straightforward challenge: given that φ is sufficient for *a*’s being *F*, why take φ + to be adequate to the explanatory task when φ is not? The claim that *F*ness is projected seems to introduce an extra explanatory step where none is needed. The simpler theory is the one that says that φ is explanatorily sufficient for *a*’s being *F*. And note that this is *especially* so if Edwards is right that the property’s being projected would entail that truth in the relevant discourse must be non-representational (see Sect. 2.2.3), for then adding in this (seemingly redundant) explanatory step would also engender a bifurcation in the nature of truth. The critical point is that falling back on φ, rather than φ+, is perfectly compatible with φ giving us a response-dependent, or otherwise anti-realist, metaphysics of *F*ness. But unless there are projected properties in Edwards’s sense, the argument for truth pluralism cannot get up and running.

#### From abundance to projection?

Despite its central place in his argument for pluralism, Edwards gives little defence of projected properties per se, because he thinks the distinction between objective and projected properties ‘broadly correspond[s] to the distinction between sparse and abundant properties’ (68). This is striking. Unlike Edwards’s rather idiosyncratic, linguistically-mediated conception of projection, the distinction between sparse and merely abundant properties is reasonably well-entrenched in contemporary metaphysics. If Edwards is right that these distinctions align, and can thereby root his argument for truth pluralism in this less controversial ontological distinction, truth pluralism will stand on much firmer ground. In open-minded spirit, then, it’s worth considering Edwards’s reasons for endorsing this alignment.

First, then: what is the distinction? On an abundant conception of properties, any (consistent) predicate whatsoever refers to a property, no matter how gerrymandered those entities in its extension. If we define the predicate ‘is quurkey’ as: for any *x*, *x* is quurkey iff (*x* is a quark or *x* is a turkey), then ‘is quurkey’ refers to a property (quurkeyness); despite the fact that, intuitively at least, there is nothing substantive and interesting that just the quarks and turkeys have in common. On a sparse conception of properties, by contrast, properties ground genuine similarities between entities and are of causal-explanatory significance. Being a quark is a property, as is being a turkey; but quurkeyness, no. One problem with a sparse conception of properties, for Edwards, is that we must deny that some predicates refer to properties, which leaves them in need of a semantic value. But on an abundant conception of properties, we cannot make sense of the difference between a causal-explanatorily significant property like being a quark and a gerrymandered property like being quurkey. Thus Edwards (35), taking his lead from Lewis (1983), endorses a pluralistic approach. Any predicate refers to a property, but a proper subset of such properties is privileged, grounding genuine similarity relations, and so on. The latter are the “sparse” properties, while the rest are “merely abundant”.

It’s certainly not apparent at first sight that this distinction does, in fact, “broadly correspond” to Edwards’s own distinction between objective and projected properties. If Gobbles is quurkey, then the complete explanation of why he is so is that he is a turkey (or that he is a quark, if he is a quark). There’s no explanatory role for falling under ‘quurkey’ to play here. So why think that merely abundant properties are projected?

We’ve thus far said nothing about what sparse and abundant properties *are*, except that sparse properties ground genuine similarities and are causal-explanatorily significant, while abundant properties do/are not. Edwards (37) is neutral on whether sparse properties are best understood as universals, tropes, or natural classes. But when it comes to abundant properties, he foregrounds “predicate nominalism” (34–35):

On this view, there are properties insofar as there are extensions of predicates: to have the property of being red is to be in the extension of ‘red’ […] On this view of properties, it is not the case that an object is in the extension of ‘is F’ because it has the property of being F; rather the object has the property of being F because it falls under the predicate ‘is F’.

According to predicate nominalists, all *and only* the properties in existence are those that are referred to by our predicates. And it certainly seems that, to avoid this being a massive and ever-evolving coincidence where properties conveniently pop in and out of existence as which predicates we have changes over time, we may well be tempted by the projective reading of (P)—an object *a* is *F because* it falls under the predicate ‘is *F*’—as Edwards suggests (the considerations at the end of Sect. 2.2.1 notwithstanding). So, if we endorse predicate nominalism as a theory of abundant properties, then it looks like merely abundant properties are, in fact, projected.

This is an intriguing move. While this is not the place to settle the metaphysics of properties,^16^ we ought to make the following three observations. First, I’ve tried to motivate Edwards’s move from predicate nominalism to projection by pointing out that the perfect alignment the predicate nominalist postulates between which properties there are and which predicates we have threatens to be incredible unless we endorse the right-to-left explanatory reading of the biconditional (P). However, some predicate nominalists might reject this move by denying that there is anything to explain. If to exemplify a property *F just is* to fall within the extension of the predicate ‘*F*’, then arguably the relevant instances of the biconditional are *trivial*: to say that *a* exemplifies the property of *F*ness just is to say that *a* falls within the extension of ‘*F*’.^17^ Second, note that the paradigm instances of merely abundant properties are gerrymandered properties like quurkeyness. It is at least unusual to think that all the properties we talk about in a particular domain—like mathematical properties, ethical properties, or socially constructed properties—are all like this. Is the pluralist committed to thinking that moral goodness is like quurkeyness, grouping together a hodgepodge of things that share no genuine similarity? (Edwards, in fact, has his own reasons for drawing such an alignment—see fn. 25.) If not, then in rooting projection—and hence truth pluralism—in merely abundant properties, Edwards may fail to vindicate the intuitions that draw many to the view in the first place.

Third, when it comes to the metaphysics of abundant properties, predicate nominalism is not the only game in town (as Edwards notes in a footnote to the passage quoted above). The class nominalist, for instance, maintains that any class of entities share a property: abundant properties are classes. An object can be a member of the class of quarks and turkeys without our having a predicate ascribing the property; as, indeed, all turkeys and quarks were until quite recently. In which case, the alignment between abundance and projection breaks down.

Now, while at various points Edwards seems to have predicate nominalism in particular in mind, he talks in general (e.g., 86) as though *any* conception of abundant properties will entail that they are projected. Indeed, in a striking footnote when he introduces the objective/projected distinction, Edwards (68) suggests that abundant properties are projected even if:

we are thinking about properties as classes, and classes as mind-independent. This is because, even if there is a vast number of classes, we still need to make sense of a predicate selecting a *particular* class, and thus having the particular extension it does, which will be dependent on our practices.

But the reasoning here is difficult to follow. As mentioned above, when it comes to predicates and properties, one salient class of questions is *metasemantic*: we can ask of any particular predicate why it refers to the property it does (‘magnetic’ to being magnetic; ‘quurkey’ to quurkeyness). If this is what Edwards means by ‘mak[ing] sense of a predicate selecting a *particular* class’, then he is correct that the answer to *this* question will mention our practices. But this must be kept separate from the question of why an object exemplifies that property: why iron is magnetic, or Gobbles is quurkey. That we explain the meaning of ‘quurkey’ in terms of our practices does not make Gobbles’s being quurkey dependent on our practices, any more than our doing so for ‘magnetic’ makes iron’s being magnetic dependent on us. The answers *might* be connected—for instance, if predicate nominalism is true—but we cannot presuppose this.

In sum, then: Edwards’s grounds for endorsing projected properties comes from a certain, highly controversial, metaphysics of abundant properties: predicate nominalism. In the spirit of generosity, let’s grant this.

#### The argument

Having found grounds of at least some kind for Edwards’s distinction between objective and projected properties, we can turn—finally—to the ensuing argument for truth pluralism.

For the sake of illustration, let’s suppose: (i) that *being magnetic* is an objective property and that a bit of iron—call it ‘Irene’—exemplifies this property; and (ii) that *being cool* is a projected property and that a motorbike—call it ‘Jeff’—exemplifies this property. The argument to truth pluralism is then as follows (84–88). Suppose all instances of the following schematic biconditionals are true:

(FT): The object referred to by ‘*a*’ falls under the predicate ‘*F*’ iff ‘*F*’ is true of the object referred to by ‘*a*’
(TO): ‘*F*’ is true of the object referred to by ‘*a*’ iff ‘*a* is *F*’ is true
(TP): ‘*a* is *F*’ is true iff the object referred to by ‘*a*’ has the property referred to by ‘*F*’

Irene has the property referred to by ‘is magnetic’. Since being magnetic is objective, by (P) this explains why Irene falls under the predicate ‘is magnetic’. Given (FT), this in turn explains why ‘is magnetic’ is true of Irene; and given (TO), this explains why ‘Irene is magnetic’ is true. Assuming the transitivity of explanation, ‘Irene is magnetic’ is thus true because the object referred to by ‘Irene’ has the property referred to by ‘is magnetic’. This is an instance of (TP), with explanatory dependence from left-to-right:

(TPi): ‘Irene is magnetic’ is true because the object referred to by ‘Irene’ has the property referred to by ‘is magnetic’

By contrast, Jeff has the property referred to by ‘is cool’. Since being cool is projected, by (P) this must be because Jeff falls under the predicate ‘is cool’. This leaves us with the question of *why* Jeff falls under the predicate ‘is cool’. Given (FT), Edwards suggests we appeal to ‘is cool’ being true of Jeff; in turn, given (TO), Edwards suggests that ‘is cool’ is true of Jeff because ‘Jeff is cool’ is true. Given the transitivity of explanation, we can thus say that the object referred to by ‘Jeff’ has the property referred to by ‘is cool’ because ‘Jeff is cool’ is true. This is another instance of (TP), but this time with the explanatory dependence from right-to-left:

(TPii): The object referred to by ‘Jeff’ has the property referred to by ‘is cool’ because ‘Jeff is cool’ is true

We can therefore read a different order of explanatory dependence into (TP) when the property is objective (left-to-right) and when it is projected (right-to-left). So, what is true is sometimes grounded in what is so, and what is so is sometimes grounded in what is true. In particular, the truth of ‘*a* is *F*’ depends on how things stand with *a* and *F*ness when *F*ness is objective, suggesting a representational conception of truth. By contrast, how things stand with *a* and *F*ness depends on whether or not ‘*a* is *F*’ is true when *F*ness is projected, suggesting a non-representational conception of truth. Thus, if some objects are objective and others are projected, then truth is sometimes representational, and sometimes non-representational.

Clearly the crucial move in this argument is from the claim that Jeff is cool because he falls under the predicate ‘is cool’, to his being cool because ‘Jeff is cool’ is true, via (FT) and (TO). Edwards (86) goes so far as to suggest that the idea that truth ‘is dependent on predicate satisfaction, is compatible only with the [objective] conception of properties… and not with the [projected] one.’ This is, at best, hasty. Granted, by definition we cannot explain why Jeff falls under ‘is cool’ in terms of Jeff’s being cool, since we’re assuming coolness is projected. But for all that’s been said there may be some other explanation (as Edwards (88) seems to concede in a footnote). This becomes pressing when we consider the limitations of Edwards’s proposal. For instance, Edwards requires that we have a singular term for every entity *x* in the extension of ‘is cool’, otherwise there is no relevant sentence of the form ‘*x* is cool’ to be true. This looks implausible, but it’s not obvious how we’re to avoid the difficulty without taking on new, substantive assumptions about truthbearers. For instance, we might shift from sentences to propositions, conceived of as structured entities composed of necessarily existent abstract objects like Fregean senses. We need details—such a move is non-trivial. However, I raise this worry only to set it aside.

The central difficulty for this line of argument is that Edwards faces an analogous challenge to the one we raised for Cotnoir and Edwards above. By his own lights, the truth of ‘Jeff is cool’ must be understood in non-representational terms; say in terms of superassertibility. But then there is a rival, non-alethic explanation of why Jeff falls under ‘is cool’ on the cards: we can say that it is because ‘Jeff is cool’ is superassertible, rather than because 'Jeff is cool' is true. Again, the specifically *alethic* element in Edwards’s explanatory story looks explanatorily idle. The rival proposal lets the property that is, by the pluralist’s own lights, co-extensive with truth in the relevant cases play the relevant explanatory role; and in doing so does not demand a bifurcation in the nature of truth. If we explain Jeff’s falling under ‘is cool’ in terms of ‘Jeff is cool’ being superassertible, then—as with objective properties—we can explain why ‘is cool’ is true of Jeff and hence why ‘Jeff is cool’ is true via the relevant instances of (FT) and (TO). That *F*ness is projected is thus perfectly compatible with ‘*a* is *F*’ being true in a representational sense.

So, even if we grant predicate nominalism about merely abundant properties, *and* the argument from predicate nominalism to projection, *and* we think that the properties of the relevant domain are merely abundant, we do not here have a compelling argument for truth pluralism.

### Conclusion to Sect. 2

In this section, we’ve considered three “ontological” reasons for thinking that while what is true sometimes depends on what is so, what is so sometimes depends on what is true, and thus that truth is sometimes representational and sometimes non-representational: that different entities literally exist in different ways; that some entities are objective while others are projected; and that some properties are sparse while others are merely abundant. While not hopeless, the arguments are unpersuasive: it is quite possible to give an anti-realist gloss on the relevant entities without having to think that their existence depends specifically on the truth of various sentences—indeed, by the pluralist’s own lights the latter just seems to introduce an extra, otherwise redundant step into the explanation—and without this specifically alethic element in the explanatory story, the argument for truth pluralism cannot get off the ground.

## Truth and causation

Our attempts to find an ontological motivation for truth pluralism have thus far been unsuccessful. But notice that we have been focusing exclusively on the purported *relata* of the correspondence relation, to the exclusion of the relation itself. (I’ll use ‘correspondence’ as a label for the relation that holds between a truthbearer and “the world” when the former is true in a substantive, representational sense.) This is dialectically significant. If correspondence ought to be understood a certain way, then this may set constraints on what can stand in the relation, thus restricting the theory’s potential scope. This is the line pursued by Lynch (2009).^18^ In particular, Lynch argues from (a) a *causal*-*representational* interpretation of correspondence, and (b) the claim that causal representationalism does not apply to certain beliefs, to argue that (c) truth for the relevant beliefs is non-representational.

In this section, we’ll consider this alternative argumentative strategy. We’ll proceed as follows. In Sect. 3.1, we’ll set out Lynch’s argument. In Sect. 3.2, we’ll consider a variety of ontological grounds for (b). Finally, Sect. 3.3 evaluates, and articulates three objections to, Lynch’s argument. Ultimately, we’ll see that we run into similar difficulties to those above. While the causal-representational conception of correspondence is effective in setting constraints on its scope, the restriction itself appears unmotivated: in particular, the pluralist appears to be committed to the adequacy of a non-causal interpretation of correspondence in just those cases where the causal interpretation gives out. Moreover, as far as I can see this issue is structural, and will therefore affect any argument for pluralism that proceeds along these lines.

### Causal representationalism

Lynch focuses on beliefs composed of concepts, which we’ll denote with angle brackets: <furry> is the concept of furriness.^19^ According to Lynch (22–32), the correspondence theory of truth finds its most plausible contemporary guise in causalist interpretations of what have been called “building block” theories of representation. Such a theory will tell you what it is for <Felix> to represent Felix, and <furry> to represent furriness. The representational content of the belief that Felix is furry is then a function of these components: it is true iff the object <Felix> represents exemplifies the property <furry> represents. Lynch (25) gives us a toy version of a causalist interpretation of such a theory, which I’ve adapted for our example:

CAUSAL: <furry> denotes furriness = instances of furriness cause, under appropriate conditions, mental tokenings of <furry>

Causal theories of representation are familiar from the literature. Just as a photo might represent Amy rather than her identical twin Annie, not because of any relevant similarity between the photo and Amy that does not hold between the photo and Annie, but because Amy was causally involved in the production of the photograph in the right way (Stampe 1977: p. 43), so too <furry> represents furriness because furriness is appropriately involved in the production of thoughts involving <furry>. Such is the broad idea.^20^ Call this, the *causal*-*representational* theory of correspondence.

CAUSAL constrains what can stand in the correspondence relation, so understood: for the belief that *a* is *G* to correspond to reality requires that the concept <*G*> is causally responsive to *G*s, so *G*s must be the type of thing with which something can causally interact. Lynch’s driving thought is that CAUSAL, and thus the correspondence conception of truth, is hereby limited in scope:

‘[…] where responsiveness is not plausible – either because the [mental] states in question aren’t appropriately causally responsive or because the external environment contains no Gs that can be so causally responsive – then it is less likely that mental-states with Gish content have that content because they represent Gs. Some other explanation of their content becomes more likely. And – to anticipate the central lesson – if we nonetheless wish to maintain that the relevant mental states are *true*, some other account of what makes them true must be pushed onto the field.’ (33)^21^

Note that, in the present context, mere scepticism about the existence of some entity (e.g. numbers)—that is, the contention that there are no *G*s—is not the kind of thing we’re after.^22^ I’m assuming that we do not want to simultaneously hold that ‘7 is prime’ is true and so 7 is prime, and that the number 7 does not exist. What we’re after are reasons for thinking the relevant entities ‘cannot be so causally responsive’. If our beliefs in some domain are concerned with entities that cannot enter the relevant causal interactions, and yet (some of) our beliefs are true, then—since correspondence is causal—we must understand truth in the relevant discourse non-representationally.

Lynch thus suggests an argument from (a) a causal-representational interpretation of correspondence, and (b) the claim that causal representationalism does not apply to certain beliefs, to (c) truth for the relevant beliefs being non-representational. (The background assumptions required to get us to truth pluralism—which we’ll grant—are that truth must at least sometimes be understood representationally, and that at least some of our (atomic) beliefs in the (b) discourses are true in a substantive sense.)

So, we’ll first (Sect. 3.2) consider four ontological grounds for (b): that certain beliefs concern entities that are (i) merely abundant; (ii) non-natural; (iii) abstract; or (iv) mind-dependent. The case is more convincing for (i)–(iii) than for (iv). We’ll then (Sect. 3.3) ask whether CAUSAL’s limited scope does, in fact, give us good reason to go in for truth pluralism.

### Causally impotent entities

#### Abundant, non-natural, and abstract entities

The case for merely abundant, non-natural, and abstract entities falling outside the scope of CAUSAL is straightforward, since in each case it follows almost by definition. Merely abundant properties, recall, we introduced in contrast to “sparse” properties, which ground genuine similarities between entities and hence are causal-explanatorily important. Something’s being a turkey predicts and explains various things about what it’s like and how it behaves. Its being quurkey does not do so, precisely because there is nothing substantive that just the quarks and turkeys have in common. While Lynch doesn’t consider merely abundant properties, if the distinction is in good standing, then they are a good example of the type of thing that might fall outside the scope of CAUSAL.

Lynch (34) seems to raise the point about non-natural entities almost in passing. When discussing moral properties, he says that ‘it is difficult to know how wrongness—even if we grant that it is a property—can be a natural property with which we can interact.’ The thought seems to be that a moral property must be *non*-natural, as per moral non-naturalism.^23^ Very roughly, non-naturalists argue that moral properties are not subject to investigation by the natural sciences, and are not reducible to such “natural” properties, but are rather autonomous entities of their own kind. The further relevant thought is that they are thus not part of the causal order of things (otherwise they *would* be subject to such investigation).

On the face of it, this is a surprising place to look for motivation for truth pluralism, since it would be odd to combine moral non-naturalism with a non-representational theory of moral truth. On the one hand, non-naturalists are traditionally the arch moral realists, so it would be surprising to see them reject a correspondence conception of moral truth. On the other, one might have hoped that going non-representational about moral truth would enable us to do without postulating such exotic entities. Still, that a partnership is surprising does not render it unworthy of consideration.

Finally, as Lynch (34) notes, abstract entities, in contrast to concrete entities, are standardly taken to lack spatiotemporal location and hence to be causally inefficacious. Paradigm abstract entities, like numbers, hereby straightforwardly fall outside the scope of CAUSAL.

One might take issue with any of the foregoing, arguing that gerrymandered, non-natural, or abstract entities are causally efficacious (and this would, in the end, be grist to my mill). However, it’s plausible enough that such entities fall outside the scope of CAUSAL that we’ll grant it for the sake of argument, to see what follows vis-à-vis pluralism.

#### Mind-dependence

Another distinction that Lynch—and, indeed, many other pluralists—brings up in this context is that between mind-independent and mind-dependent entities. According to Lynch (33), naturalistic theories of representation like CAUSAL, and even its non-naturalistic predecessors, are committed to:

‘[t]rue beliefs map[ping] objects that exist and have their properties mind-independently. […] An object exists (or has some property) mind-independently at some time just when it would continue to exist (or have that property) even if there were no minds that represented it as having that property.’

He thus argues that, for instance, “legal propositions” fall outside the scope of CAUSAL. But why think that CAUSAL is committed to the mind-independence of the “worldly” relatum? In its defence, Lynch (33) only says that it is ‘a consequence of the fact that representational views intend their position to be realist.’ So construed, however, it is an additional bolt-on, and not a consequence, of the view. (Besides, I’m sceptical that mind-independence is necessary for “realism”, in any non-stipulative sense—see e.g. Barnes (2017).)

Why think that mind-dependence frustrates CAUSAL? One thought might be that mind-independence is necessary for causal efficacy. But it would be, at best, surprising to find out that all mind-dependent properties and facts are causally redundant. Is Prince William’s inheritance of Catherine Middleton’s estate in the event of her death not caused by his being married to her? Is someone’s being fined for speeding not caused by the fact that it is illegal? Many think that race and gender are mind-dependent (perhaps because “socially constructed”); but presumably still want to say that someone’s race or gender might cause them to be subject to various kinds of structural privilege or oppression. (One might respond that these are not instances of causation, but of non-causal explanation. But to do this merely on the basis of mind-dependence seems ad hoc.)^24^

One may concede that mind-dependent entities enter into causal relations while thinking that there is a difference in the *kind* of causal role we attribute to them. Take, for instance, Wright’s (1992: p. 196) notion of “width of cosmological role”. A state of affairs has a “wide” or “broad” cosmological role only if it is ‘potentially contributive to the explanation of things *other than*, or *other than* via, our being in attitudinal states which take such states of affairs as object.’ One might suspect that mind-dependent states of affairs have only narrow cosmological role. For instance, that Prince William inherits Catherine Middleton’s estate seems to be explained by the fact that the right people *believe* he is married to her. If all the explanatory work of such states of affairs, and hence the causal work, goes “via” our mental states about them, then it has only a “narrow” cosmological role. By contrast, that iron behaves the way it does around magnets is explained in terms of its being magnetic, without any intermediary reference to our mental states; so iron’s being magnetic has “wide” cosmological role.^25^ However, even if mind-dependent states of affairs *ipso facto* have narrow cosmological roles, this does not automatically have any bearing on CAUSAL, since it is explicitly built into the definition of a narrow cosmological role that such states of affairs *can* (directly, as it were) explain ‘our being in attitudinal states which take such states of affairs as object’.^26^ If this explanation is not to be construed as a causal explanation in the case of mind-dependent states of affairs, we need to be told why not.

In that spirit, let me offer a tentative reconstruction of why one might take mind-dependence to be incompatible with CAUSAL. As with the arguments considered in Sect. 2, the principal issue concerns explanatory circularity. Suppose that instances of marriage are grounded in our mental states; in particular, that whether or not someone is married depends on whether or not (certain relevant) people *believe* that they are married. (Note that this is a stronger condition than Lynch’s mind-dependence, since it appeals to *particular* mental states.) Now, such beliefs are partially individuated by the fact that they are *about marriage*: that <marriage> represents marriage. If we in turn appeal to CAUSAL to explain this representational fact, we appeal to instances of marriage to explain why this belief is about marriage. But instances of marriage are meant to be grounded in beliefs about marriage; and these beliefs about marriage will need to be explained in terms of instances of marriage… And so on, around and around. So—the reasoning goes—one cannot use CAUSAL to explain the content of concepts concerning mind-dependent properties, on pain of circularity.

As I say, I offer this reconstruction only tentatively; I am unconvinced myself. (I’m not convinced that this need be viciously circular rather than a benign regress, for example.) However, let’s grant it for the sake of argument, since I think using mind-dependence as a basis for truth pluralism makes Lynch’s position much more interesting. (One would be hard-pressed to deny that anything is mind-dependent, after all.)

### Criticism

To recap: Lynch argues from (a) a causal-representational theory of correspondence, and (b) that causal representationalism does not apply to certain beliefs, to (c) that truth for the relevant beliefs is non-representational. We’ve considered four ontological grounds for (b): that the entities the relevant discourses are concerned with are merely abundant, non-natural, abstract, or mind-dependent. In this section, I’ll raise three objections to Lynch’s argument: the first concerning (b), the second concerning (a), and the third concerning the inference from (a) and (b) to (c).

First, regarding (b), Lynch’s argumentative strategy presents a couple of problems. He makes the case for (b) by discussing CAUSAL; but CAUSAL is explicitly a toy theory, to be replaced by something more sophisticated. It’s thus a live question to what extent the critique of CAUSAL will be a reliable guide to the scope of its sophisticated successor. Stewart Shapiro (2009) makes this point with regards to <nitrogen> and <gravitational field>:

‘To belabor the obvious, nitrogen does not “cause, under appropriate circumstances, mental tokenings of” [<nitrogen>]. […] For one thing, we are (almost) always in the presence of nitrogen. Similarly, mental states with gravitational-field-ish content are not causally responsive to an external environment that contains gravitational fields. *Every* external environment contains a gravitational field.’^27^

One option, of course, is to incorporate discourses that use such “theoretical” concepts into (b), and thus place them outside the scope of correspondence. I take it this would make the resultant pluralism less attractive to many. But Shapiro’s point is a dialectical one: that the sophisticated theory may well capture such concepts, rendering CAUSAL an unreliable guide. As he puts it: ‘There may nevertheless be a scope problem for the envisioned correspondence based property […] but we’ll have to wait for details to find out what it is.’

More importantly, once reference has been secured for *some* concepts, there is a natural *extension* of the program to secure reference to further entities. Consider <quurkey> again. Suppose that <quark> and <turkey> refer to quarks and turkeys, respectively, by virtue of standing in the right causal relation to quarks and turkeys. Then we can define the concept of quurkeyness as before: *x* is quurkey iff (*x* is a quark or *x* is a turkey).^28^ In this way, the causal account can provide a kind of “source intentionality”, of which the intentionality of other concepts can be derivative. Indeed, the structure of this strategy is enshrined in Lewis’s (1970) proposal for “How to Define Theoretical Terms” (though Lewis is not committed to a causal derivation of source intentionality). Very roughly, given a scientific theory—about, say, nitrogen—one can “Ramsify” out the theoretical term: replace every occurrence with a variable, *x*, bound by an existential quantifier. Your “Ramsified” theory says that there is an entity, *x*, that has all the features that the theory takes nitrogen to have. Provided that the *other* terms that occur are already meaningful (in our case, through having their reference explained causally), <nitrogen> refers to the entity, supposing there is one, that satisfies the description (or the best deserver). The point is: granting causal representationalism for certain concepts, provided that your source intentionality is rich enough, one can secure reference to further entities despite the absence of the relevant causal relation. If such a strategy cannot be extended to accommodate moral, mathematical, institutional, etc. discourse, we need to be told why.

So, while it’s plausible that reference to merely abundant, non-natural, and abstract entities cannot be secured through direct causal means, it is much more contentious that such entities remain beyond the reach of a causal theory that is supplemented in this way. Merely abundant properties like quurkeyness are straightforwardly accounted for; and moral functionalists already argue that reference to moral properties is best understood in something like this way.^29^

Second, note that Lynch himself doesn’t provide much by way of argument for (a). He states that contemporary versions of the correspondence theory are ‘widely accepted in philosophy and implicitly accepted by many cognitive scientists and psychologists’ (22); and then makes a case that causal representationalism is such a contemporary version.^30^ Now, the tenor of the discussion suggests that Lynch sees causal representationalism as indispensable to ‘the over-arching research program of cognitive science [which] takes it that the mind—that is, the brain—is an organ part of whose function is to represent the world around it’ (22). But this would be a contentious claim indeed—*that* the brain represents the world around it is one issue; *how* it does so is quite another, which might admit of non-causal explanation.^31^ CAUSAL, or its sophisticated successor, may well be plausible for certain concepts; but we ought to be open to a non-causal theory of representation, at least for certain other concepts, should one be forthcoming.

Which brings us neatly to the final, principal difficulty facing Lynch’s argumentative strategy. Lynch suggests that, where representation cannot be explained in causal terms, some non-representational theory of *truth* must be appealed to. But the most this could show is that *representation* cannot always be cashed out in causal terms. It’s compatible with this that it should be explained in non-causal terms for abundant, non-natural, abstract, or mind-dependent entities; and thus that truth should always be a matter of accurate representation. That is, we might appeal to a pluralistic theory of *reference determination*, rather than truth.^32^

Lynch (162–163) is sensitive to something like this worry, but points out that coming up with such a theory “seems difficult”. But now we come full circle. By Lynch’s own lights, truth in the relevant domain consists in some non-representational property, like superassertibility.^33^ Therefore, by his own lights an extensionally adequate theory of reference determination is one that appeals to the relevant truthbearers being superassertible (rather than true). For instance, suppose that the moral belief that *x* is wrong is true iff it is superassertible.^34^ If this is right, we have at our disposal a substantive, non-causal theory of reference determination for ethical concepts: *x* is in the extension of <wrong> iff <*x*> refers to *x* and the belief that *x* is wrong is superassertible. But now, having explained *reference* in terms of superassertibility, the *truth* of the belief can be explained “building-block”-style, in the manner Lynch prefers. Coming up with a pluralistic theory of reference determination is thus no more (or less!) difficult than coming up with a pluralistic theory of truth. And if CAUSAL and its ilk count as explications of correspondence, so does this. Once again, theoretical economy endorses the theory that does not bifurcate the nature of truth *as well as* reference.

Finally, it’s worth noting that, as far as I can see, nothing in the structure of this dialectic hinges on the fact that Lynch is appealing to a *causal* theory of correspondence. I thus suspect this kind of challenge could be pressed against any argument for pluralism that goes via arguing, on ontological grounds, that your preferred representational conception of truth has limited scope.

Lynch’s attempt to motivate pluralism via a causal-representationalist theory of correspondence is therefore uncompelling. On the one hand, a sophisticated causal representationalism can in general explain reference to entities with which we do not causally interact in the correct way, meaning the scope of the theory is still an open question. On the other, the causal constraint on representation is itself unmotivated; and, indeed, pluralists themselves look to be committed to the extensional adequacy of a non-causal theory in just those cases in which the causal account fails.

## Conclusion

Drawing on material provided by pluralists themselves, we’ve surveyed a variety of potential ontological grounds for truth pluralism: that certain entities (i) literally exist in a different way; (ii) are projected; (iii) are merely abundant; (iv) are non-natural; (v) are abstract; or (vi) are mind-dependent. In such cases, the Edwards-style strategy is to argue that what is so depends on what is true; so truth must be non-representational. The Lynch-style strategy is to argue that truth is non-causal, and hence non-representational. While not hopeless, we’ve seen that neither strategy is especially compelling. The additional premises needed to move from such ontological distinctions to truth pluralism are unattractive, often only serving to bifurcate the nature of truth when more conservative options are available. This makes me pessimistic about the prospects for building truth pluralism on ontological foundations.

But even if that is right, all is not lost. The metaphysical strategies we’ve been considering start from the idea that there is an important dimension of ontological variation between the entities that different discourses are concerned with, and try to argue for truth pluralism on this basis. The inevitable question is: why not just appeal to variation in the kinds of entities that sit on one end of the correspondence relation, rather than bifurcating the nature of truth?

Contrast this starting point, however, with metaethical expressivism. Expressivists characteristically deny that we get an informative explanation of what’s going on in the moral domain by postulating a realm of moral facts or properties that we use moral talk to describe. Instead, we should postulate a set of pro- and con-attitudes—attitudes of approval and disapproval, say—that we use moral talk to express. Such states have a “desire-like” functional role, rather than a “belief-like” one: functioning to push us into action, rather than to represent the world around us. The crucial disanalogy is that the expressivist does not postulate entities of a peculiar ontological kind to explain moral discourse. Rather, she does not postulate entities for this explanatory purpose *at all*. The expressivist does not appeal to an *ontological* distinction, but a *functional* one: moral thought and talk serves a non-representational function. At the outset, then, there is no question of moral truth consisting in accurate representation of the moral facts; for there are no facts to sit on the “worldly” end of the correspondence relation.^35^ The version of “double-counting” worry we’ve been considering thus cannot get a grip.

Alas, expressivists have not tended to be truth pluralists. Early expressivists denied that moral discourse is truth-apt^36^; contemporary expressivists, especially “quasi-realists”, typically go in for deflationism.^37^ But while I cannot argue it here, I suspect that there are good reasons for expressivists to be dissatisfied with a deflationary conception of moral truth.^38^ Truth pluralists would be well-served if so. The third way between deflationism and correspondence is to appeal to a substantive, but non-representational, conception of moral truth. If we must nonetheless go in for a representational conception of truth elsewhere—such as in discourses that *do* serve a representational function—then the result is truth pluralism; one grounded in an underlying functional or teleological diversity, rather than an ontological one.

Moreover, I’d be surprised to find that those sympathetic with a non-representational conception of truth in a particular discourse were not also attracted to a non-representational explanation of the discourse’s function. Indeed, this seems a more natural home for truth pluralism than one which sees such discourses as uniformly representational in function, but concerned with different kinds of entity. Of course, it remains to be seen if such a strategy is viable, or if it runs into “double-counting” worries of its own. But given what I’ve argued here, pluralists ought to be interested in finding out.

## Footnotes

1 See Dorsey (2006), Lynch (2009: Chap. 8), and Gamester (2017: Chap. 5) for cognate suggestions.

2 Throughout this paper, my focus is on “inflationary” pluralisms that endorse both representational and non-representational truth properties, which is the locus of most of the philosophical attention pluralism has garnered thus far. Rather than: “correspondence” pluralisms, which endorse only representational truth properties (e.g. Sher 1998); hybrid pluralisms, which endorse inflationary and deflationary truth properties (McGee 2005; Kölbel 2008; Ferrari and Moruzzi 2018); and deflationary pluralisms, which endorse only deflationary truth properties (Beall 2013). Considering other kinds of pluralism would, unfortunately, complicate the dialectic substantially; nonetheless, other pluralists may well find help or hindrance within.

3 Besides the work discussed below, only Pedersen (2014) and Ball (2017) have engaged seriously with the question of the underlying motivations for pluralism.

4 One could try to defuse this worry by arguing that the existential quantifier receives a “deflationary” reading in such cases. This does not affect the dialectical point I am making here, since to do so is to move beyond the kind of rough-and-ready, intuitive motivation with which we started, and instead draw on substantive, philosophically interesting contrasts between domains—just as I’m suggesting we should.

5 The paradigm here is metaethical, or metanormative, expressivism: ‘although the teleology of spatial perception is spatial, the teleology of ethical commitment is not ethical.’ (Blackburn 1993: p. 169).

6 Nor need they be exhaustive. Perhaps further strategies could appeal to, say, epistemological or normative differences (perhaps including Wright’s (1992) cruces: evidence-transcendence, cognitive command, width of cosmological role, etc.). However, I’m inclined to think that such differences between discourses will likewise demand explanation, and that the natural explanatory strategies are, once more, ontological or teleological. If so, then while epistemological or normative differences may be an important part of an ontologically- or teleologically-driven strategy, ontology or teleology will be explanatorily fundamental.

7 On normative differences in this context, see e.g. Ferrari (2019), Pedersen (2020).

8 Though Lynch (2013, 2015, 2019) seems to be shifting towards the teleological strategy (see also Wright 1998: p. 191). Other exceptions are Ball (2017) and Gamester (2017).

9 See Quine (1960: pp. 118–119); Sainsbury (1996); Dodd (2013); Asay (2018); Bar-On and Simmons (2019). Unlike some of these presentations, I’ve tried to meet pluralists more on their own terms. (I do not, for instance, assume the adequacy of a deflationary conception of truth or truthmaker theory.) Let me hereby cancel any implication that the teleological strategy doesn’t face an analogous challenge—see Blackburn (2013).

10 Nikolaj Pedersen also suggests that truth pluralism should be partly founded in a kind of “metaphysical pluralism”—see especially Pedersen (2014). However, since his goal is only to show the latter ‘fits well’ with the former, rather than to provide anything like an argument (2014: p. 271), I have not found it productive to engage directly with Pedersen’s suggestive work here.

11 The dilemma does not appeal to any peculiar features of *sentences* qua truthbearers, so would presumably recur if we subbed in propositions or whatever else instead. At this, but only this, point in the paper, the authors seem to take (I) to be essential to ontological pluralism, in which case the dilemma threatens refutation. But it seems dispensable.

12 It’s interesting that, if the argument worked, strictly it would only show that *some* mathematical sentences are true in a non-representational sense.

13 All citations in this section to Edwards (2018), unless otherwise noted.

14 The fact that motorbikes fall within the extension of ‘is cool’ is pretty cool, simply because the phenomenon of linguistic representation is pretty cool. But that isn’t what makes motorbikes themselves cool.

15 According to Edwards (67–69), moral expressivists that are happy to say that there are moral properties, like Blackburn (1993: p. 181) and Gibbard (2006), are committed to such properties being projected. On the contrary, expressivists are typically at pains to argue that expressivism doesn’t entail that morality is mind-dependent (let alone language-dependent).

16 Predicate nominalism faces serious worries (e.g., Armstrong 1978: Chap. 2; Edwards 2014: Chap. 5.2) but (i) so do other theories of properties; and (ii) these are normally levelled against predicate nominalism as a monistic theory of properties, rather than as part of a pluralistic theory. So I won’t bother running through independent objections here.

17 Of course, if trivial then the relevant instances of (P) are incompatible with an objective reading too. So on this approach Edwards’s taxonomy of properties threatens to be non-exhaustive.

18 All citations in this section to Lynch (2009), unless otherwise noted.

19 Lynch primarily talks in terms of beliefs, only sometimes propositions. He also suggests (23) that the considerations apply *mutatis mutandis* to sentences.

20 Lynch (26) also considers a toy teleosemantic theory, but its implications for our purposes are no different from CAUSAL.

21 See also Lynch (2001: p. 724; 2004: p. 285). By ‘the [mental] states in question aren’t appropriately causally responsive’ to the external environment, I suspect that Lynch has partly in mind the contention that takes centre-stage in Lynch (2013): that thought in some domains in non-representational. This would form part of a functional or teleological, rather than ontological, motivation for pluralism.

22 Though contrast Lynch (2008: p. 122).

23 ‘Natural’ is used in many ways in the literature, so perhaps this is not what Lynch had in mind. But the suggestion is pertinent nonetheless.

24 For this reason among others, it is perplexing that mind-dependence is so often alluded to as an obvious motivating factor for truth pluralism, e.g. Cotnoir and Edwards (2015: p. 118); Edwards (2011: p. 29); Lynch (2001: p. 724; 2004: p. 385); Pedersen (2006: p. 102; 2010: p. 93); Wright and Pedersen (2010: p. 210). Note that I am *not* here suggesting that all, say, moral, institutional, or mathematical explanations are causal explanations. All I’m objecting to is the idea that we can infer that certain explanations are non-causal on the grounds that the relevant entities are mind-dependent.

25 Width of cosmological role plays a prominent role in Edwards’s overall theory: he (2018: p. 70) suggests that narrow cosmological role is a sign that a property is merely abundant (and thus projected; meaning truth in the relevant discourse is non-representational). Even if this is right, it would not affect the earlier arguments.

26 One could maintain that some states of affairs have only *super-narrow* cosmological role, which rules this out. Then, it seems, CAUSAL becomes straightforwardly circular. (This is one way of reading Lynch’s (161–162) argument concerning moral properties.) This is risky. One might reasonably think that error theory or fictionalism is more appropriate for such states of affairs: *being a witch* has a super-narrow cosmological role because there are no witches.

27 See also Smith (2010) and Connolly (2012).

28 This requires that the logical concepts involved in the definition are also meaningful. This is important, but creates complications we need not get hung up on here.

29 E.g. Jackson (1998). While functionalism is typically employed by naturalists, the strategy itself is compatible with non-naturalism.

30 I am willing to grant this for the purposes of this paper. But one might think of CAUSAL as a theory of meaning or content, *rather than* truth. For instance, one might argue that CAUSAL is a theory about how a proposition comes to be the content of a particular mental state (or expressed by a particular sentence); while a theory of truth should tell us what it is for that proposition to be true.

31 Inferentialist and interpretationist competitors spring to mind. Williams (2020) argues that the latter can concede that, and even explain why, causalists and inferentialists latch onto informative generalisations in certain cases, thereby explaining the local attraction of the view that Lynch (e.g. 2009: p. 33) leans on.

32 As Lynch (34, fn. 16) seems to concede. This is Shapiro’s suggested modification; see also Sher (1998). We might think of the extension just discussed as introducing such a bifurcation: reference determination for source intentionality is causal; otherwise it’s descriptive.

33 For Lynch (171–177), it is a property he calls *concordance*.

34 Superassertibility applies more naturally to sentences than beliefs; but the point I’m making is structural.

35 However, see Ridge (2014) for an ambitious attempt to “earn the right” to a correspondence conception of moral truth on expressivist grounds.

36 Ayer (1936).

37 Blackburn (1993) and Gibbard (2003).

38 Three principal difficulties concern: (i) accounting for first-personal moral fallibility (that is, the possibility that my own moral judgements might be incorrect) given that we’re not to postulate moral facts to determine (in)correctness; (ii) earning the right to truth-conditional semantics for moral discourse; and (iii) the problem of “creeping minimalism” (Dreier 2004). Given a deflationary conception of truth, the notion appears too thin to help the expressivist make sense of a gap between what she takes to the be case, morally speaking, and what is in fact the case, morally speaking; to explain the meaning of moral sentences; or to distinguish quasi-realism from realism. Indeed, despite his deflationary sympathies, the resources Blackburn uses to explain moral fallibility are strongly reminiscent of Wright’s superassertibility—a connection Blackburn (1993: 22, fn. 10) himself explicitly draws. However, these are large, complicated issues.

## References

[CR1] Armstrong D (1978). Nominalism and realism: Volume 1: Universals and scientific realism.

[CR2] Asay J (2018). Putting pluralism in its place. Philosophy and Phenomenological Research.

[CR3] Ayer A (1936). Language, truth and logic.

[CR4] Ball B (2017). Alethic pluralism and the role of reference in the metaphysics of truth. Southern Journal of Philosophy.

[CR5] Barnes E (2017). Realism and social structure. Philosophical Studies.

[CR6] Bar-On D, Simmons K, Pedersen N, Wyatt J, Kellen N (2019). Truth: One or many?. Pluralisms in truth and logic.

[CR7] Beall J, Pedersen N, Wright C (2013). Deflated truth pluralism. Truth and pluralism: Current debates.

[CR8] Blackburn S (1993). Essays in quasi-realism.

[CR9] Blackburn S, Pedersen N, Wright CD (2013). Deflationism, pluralism, expressivism, pragmatism. Truth and pluralism: Current debates.

[CR10] Connolly N (2012). Truth as, at most, one. International Journal of Philosophical Studies.

[CR11] Cotnoir A, Edwards D (2015). From truth pluralism to ontological pluralism and back. Journal of Philosophy.

[CR12] Dodd J, Pedersen N, Wright CD (2013). Deflationism trumps pluralism!. Truth and pluralism: Current debates.

[CR13] Dorsey D (2006). A coherence theory of truth in ethics. Philosophical Studies.

[CR14] Dreier J (2004). Meta-ethics and the problem of creeping minimalism. Philosophical Perspectives.

[CR15] Edwards D (2011). Simplifying alethic pluralism. Southern Journal of Philosophy.

[CR16] Edwards D (2014). Properties.

[CR17] Edwards D (2018). The metaphysics of truth.

[CR18] Ferrari F, Pedersen N, Wyatt J, Kellen N (2019). Normative alethic pluralism. Pluralisms in truth and logic.

[CR19] Ferrari F, Moruzzi S (2018). Ecumenical alethic pluralism. Canadian Journal of Philosophy.

[CR20] Gamester, W. (2017). *The diversity of truth*: *A case study in pluralistic metasemantics*. Ph.D. thesis. University of Leeds.

[CR21] Gibbard A (2003). Thinking how to live.

[CR22] Gibbard A (2006). Normative properties. In: Horgan, T. and Timmons, M. eds. Southern Journal of Philosophy.

[CR23] Jackson F (1998). From metaphysics to ethics: A defence of conceptual analysis.

[CR24] Kölbel M (2008). “True” as ambiguous. Philosophy and Phenomenological Research.

[CR25] Lewis D (1970). How to define theoretical terms. Journal of Philosophy.

[CR26] Lewis D (1983). New work for a theory of universals. Australasian Journal of Philosophy.

[CR27] Lynch M, Lynch M (2001). A functionalist theory of truth. The nature of truth: Classic and contemporary perspectives.

[CR28] Lynch M (2004). Truth and multiple realizability. Australasian Journal of Philosophy.

[CR29] Lynch M (2008). Alethic pluralism, logical consequence, and the universality of reason. Midwest Studies in Philosophy.

[CR30] Lynch M (2009). Truth as one and many.

[CR31] Lynch M (2013). Expressivism and plural truth. Philosophical Studies.

[CR32] Lynch M, Gross S, Tebben N, Williams M (2015). Pragmatism and the price of truth. Meaning without representation: Essays on truth, expression, normativity, and naturalism.

[CR33] Lynch M, Pedersen N, Wyatt J, Kellen N (2019). Truth pluralism, quasi-realism and the problem of double-counting. Pluralisms in truth and logic.

[CR34] McGee V (2005). Two conceptions of truth? Comment. Philosophical Studies.

[CR35] Pedersen N (2006). What can the problem of mixed inferences teach us about alethic pluralism. The Monist.

[CR36] Pedersen N (2010). Stabilizing alethic pluralism. Philosophical Quarterly.

[CR37] Pedersen N (2014). Pluralism × 3: Truth, logic, metaphysics. Erkenntnis.

[CR38] Pedersen N (2020). On the normative variability of truth and logic. Inquiry: An Interdisciplinary Journal of Philosophy.

[CR39] Quine W (1960). Word and object.

[CR40] Ridge M (2014). Impassioned belief.

[CR41] Sainsbury R (1996). Crispin Wright: Truth and objectivity. Philosophy and Phenomenological Research.

[CR42] Shapiro, S. 2009. Review of *Truth as one and many*. In *Notre Dame philosophical reviews*. <https://ndpr.nd.edu/news/truth-as-one-and-many/>.

[CR43] Sher G (1998). On the possibility of a substantive theory of truth. Synthese.

[CR44] Smith N (2010). Review of Michael Lynch, truth as one and many. Analysis.

[CR45] Stampe D (1977). Towards a causal theory of linguistic representation. Midwest Studies in Philosophy.

[CR46] Williams J (2020). The metaphysics of representation.

[CR47] Wright CJG (1992). Truth and objectivity.

[CR48] Wright CJG (1998). Comrades against quietism: Reply to Simon Blackburn on *Truth and objectivity*. Mind.

[CR49] Wright CD, Pedersen N, Wright CD, Pedersen N (2010). Truth, pluralism, monism, correspondence. New waves in truth.

